# Effect of Financial Incentives on Patient Use of Mailed Colorectal Cancer Screening Tests: A Randomized Clinical Trial

**DOI:** 10.1001/jamanetworkopen.2019.1156

**Published:** 2019-03-22

**Authors:** Shivan J. Mehta, Rebecca S. Pepe, Nicole B. Gabler, Mounika Kanneganti, Catherine Reitz, Chelsea Saia, Joseph Teel, David A. Asch, Kevin G. Volpp, Chyke A. Doubeni

**Affiliations:** 1 Department of Medicine, Perelman School of Medicine, University of Pennsylvania, Philadelphia; 2 Penn Medicine Center for Health Care Innovation, University of Pennsylvania, Philadelphia; 3 Center for Health Incentives and Behavioral Economics, Leonard Davis Institute of Health Economics, University of Pennsylvania, Philadelphia; 4 Leonard and Madlyn Abramson Cancer Center, University of Pennsylvania, Philadelphia; 5 Department of Family Medicine and Community Health, Perelman School of Medicine, University of Pennsylvania, Philadelphia; 6 Center for Health Equity Research and Promotion, Philadelphia VA Medical Center, Philadelphia, Pennsylvania

## Abstract

**Importance:**

Mailing fecal immunochemical test (FIT) kits to patients’ homes has been shown to boost colorectal cancer screening rates, but response rates remain limited, and organized programs typically require repeated outreach attempts. Behavioral economics has shown that offering salient financial incentives to patients may increase participation in preventive health.

**Objective:**

To compare the impact of different financial incentives for mailed FIT outreach.

**Design, Setting, and Participants:**

This 4-parallel-arm randomized clinical trial included patients aged 50 to 75 years who had an established primary care clinician, at least 2 visits in the prior 2 years, and were eligible for colorectal cancer screening and not up-to-date. This study was conducted at urban primary care practices at an academic health system from December 2015 to February 2018. Data analysis was conducted from March 2018 to September 2018.

**Interventions:**

Eligible patients received a letter from their primary care clinician that included a mailed FIT kit and instructions for use. They were randomized in a 1:1:1:1 ratio to receive (1) no financial incentive; (2) an unconditional $10 incentive included with the mailing; (3) a $10 incentive conditional on FIT completion; or (4) a conditional lottery with a 1-in-10 chance of winning $100 after FIT completion.

**Main Outcomes and Measures:**

Fecal immunochemical test completion within 2 and 6 months of initial outreach.

**Results:**

A total of 897 participants were randomized, with a median age of 57 years (interquartile range, 52-62 years); 56% were women, and 69% were black. The overall completion rate across all arms was 23.5% at 2 months. The completion rate at 2 months was 26.0% (95% CI, 20.4%-32.3%) in the no incentive arm, 27.2% (95% CI, 21.5%-33.6%) in the unconditional incentive arm, 23.2% (95% CI, 17.9%-29.3%) in the conditional incentive arm, and 17.7% (95% CI, 13.0%-23.3%) in the lottery incentive arm. None of the arms with an incentive were statistically superior to the arm without incentive. The overall FIT completion rate across all arms was 28.9% at 6 months, and there was also no difference by arm. The completion rate at 6 months was 32.7% (95% CI, 26.6%-39.3%) in the no incentive arm, 31.7% (95% CI, 25.7%-38.2%) in the unconditional incentive arm, 26.8% (95% CI, 21.1%-33.1%) in the conditional incentive arm, and 24.3% (95% CI, 18.9%-30.5%) in the lottery incentive arm.

**Conclusions and Relevance:**

Mailed FIT resulted in high colorectal cancer screening response rates in this population, but different forms of financial incentives of the same expected value ($10) did not incrementally increase FIT completion rates. The incentive value may have been too small or financial incentives may not be effective in this context.

**Trial Registration:**

ClinicalTrials.gov Identifier: NCT02594150

## Key Points

**Question:**

Can different forms of financial incentives (unconditional, conditional, or lottery) boost response rates to mailed colorectal cancer screening outreach?

**Findings:**

In this randomized clinical trial of 897 patients, there was no statistically significant difference in screening response rates at 2 and 6 months between the incentive arms and mailed outreach without incentive.

**Meaning:**

Different forms of financial incentives of the same expected value ($10) did not increase fecal immunochemical test completion rates, as the incentive value have been too small or financial incentives may not be effective in this context.

## Teaser

This 4-parallel-arm randomized clinical trial determines the effectiveness of financial incentives on fecal immunochemical test screening completion vs no incentive among adults who were sent kits by mail.

## Introduction

Colorectal cancer (CRC) is a leading cause of cancer death worldwide.^1,2,3,4,5^ Failure to receive or stay current on screening increases the risk of CRC death more than 2-fold.^6^ Direct mailing of a fecal immunochemical test (FIT) to patients has been shown to boost screening uptake^7,8,9,10,11,12^ but has not been widely adopted because of barriers to implementation.^13^ Response rates to mailed FIT are often limited by the reliance of the approach on resource-intensive repeat outreach. Thus, there is a need to refine the components of mailed FIT outreach to increase efficiency and response.

Colorectal cancer screening and other health promotion interventions are subject to present-time bias since the future benefits (averted diagnosis or death) are often temporally dissociated from the cost or harms of the activity.^14,15^ Additionally, patients have an additional cost of keeping track for prevention activities.^16^ Therefore, there has been interest in using behavioral economic insights to overcome such biases through patient-centered incentives.^17,18^ Financial incentives to patients have had mixed results for increasing CRC screening uptake. In one study, offering a 1-in-10 chance of winning $50 increased fecal occult blood test response rate,^19^ but in another study, a $10 incentive conditional on completing mailed FIT was not effective.^20^ In our previous work, a $100 conditional incentive plus direct access scheduling modestly increased colonoscopy use,^21^ but a decrease in cost-sharing by a much larger amount was not associated with an increase in rates.^22^

Thus, the effectiveness of financial incentives for boosting CRC screening may depend on design and context. Lotteries provide an overly optimistic expectation of winning,^23,24,25^ and unconditional incentives given upfront invoke principles of endowment and reciprocity,^26,27,28^ as compared with fixed conditional incentives that allow patients to gain a reward on completing screening. Although response rates to mailed FIT are suboptimal in many settings, it is unclear if financial incentives improve completion rates. In this study, we compare mailed FIT outreach alone with 3 incentive designs (unconditional, conditional, and lottery) of the same expected value. Our hypothesis is that financial incentives of the same expected value have different effects on uptake of mailed FIT outreach depending on how they are designed.

## Methods

### Study Design

**Figure 1.**
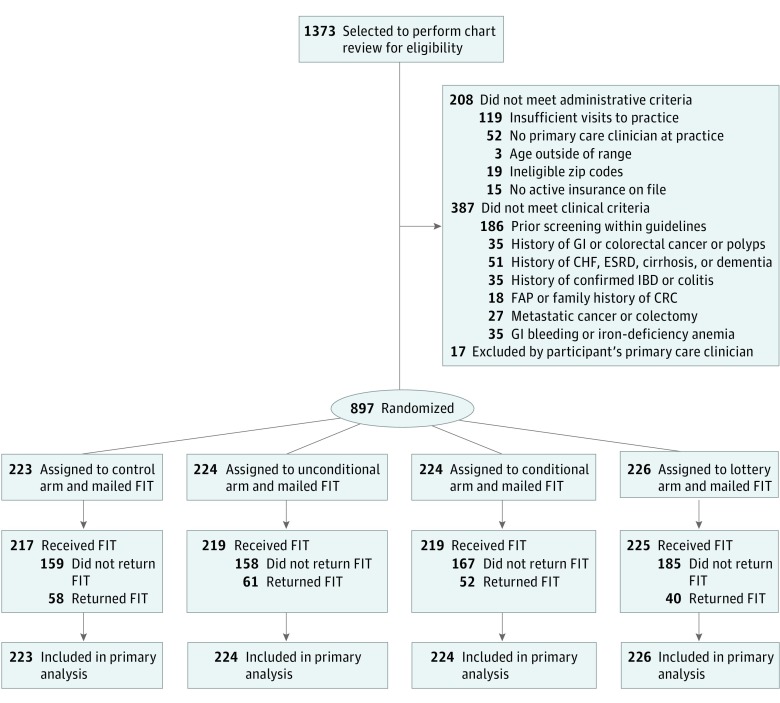
CONSORT Flow Diagram of Randomized Clinical Trial to Increase Rates of Colorectal Cancer Screening CHF indicates congestive heart failure; CRC, colorectal cancer; ESRD, end-stage renal disease; FAP, familial adenomatous polyposis; FIT, fecal immunochemical test; GI, gastrointestinal; and IBD, inflammatory bowel disease.

This was a 4-parallel-arm pragmatic randomized clinical trial of mailed FIT to determine the effectiveness of financial incentives on FIT screening completion rate vs no incentive among eligible patients who were sent kits by mail. The different forms of incentives were (1) no financial incentive; (2) an unconditional $10 incentive included with the mailing; (3) a $10 incentive conditional on FIT completion; or (4) a conditional lottery with a 1-in-10 chance of winning $100 after FIT completion. The University of Pennsylvania institutional review board approved the study. A waiver of informed consent was obtained since the protocol posed no more than minimal risk to participants and could not be practicably carried out without the waiver.^29^ The trial protocol is available in Supplement 1. This study followed the Consolidated Standards of Reporting Trials (CONSORT) reporting guideline (Figure 1).

### Study Population

The study population included patients at an academic family medicine practice at the University of Pennsylvania serving a demographically and socioeconomically diverse population in Philadelphia. Patients were identified via an automated data pull from the electronic health record (EHR) between December 2015 and July 2017. We included patients aged 50 to 75 years with at least 2 visits to the clinic in the past 2 years who were overdue for CRC screening and had a home zip code within the Philadelphia-Wilmington-Camden metropolitan statistical area. Overdue was defined as not having had a colonoscopy in the past 10 years, flexible sigmoidoscopy in the last 5 years, or stool testing in the past year. We excluded patients with a personal or family history of CRC, colonic polyps, hereditary nonpolyposis colorectal cancer syndrome, gastrointestinal cancer, gastrointestinal bleeding, iron-deficiency anemia, inflammatory bowel disease, Lynch syndrome, or familial adenomatous polyposis syndrome. Patients were also excluded if they had a diagnosis of end-stage renal disease, metastatic cancer, cirrhosis, congestive heart failure, or dementia because such conditions may compromise life expectancy and outweigh the benefits of screening. Medical record review for all patients was performed by research staff after automated data pull to confirm eligibility. For all included patients, primary care clinicians (physicians or nurse practitioners) were provided an opportunity to remove any of their patients from participation through opt-out messaging if they did not think they were appropriate for FIT outreach (eTable in Supplement 2).

### Interventions

Eligible patients were randomized in a 1:1:1:1 ratio using a computerized random number generator in 6 batches ranging from 111 to 311 to accommodate the capacity of the research staff to conduct medical record review and mail out kits. All patients received a FIT kit and a letter from their primary care clinician indicating they were receiving a FIT because they were due for CRC screening. The FIT kit contained instructions including a telephone number to call with questions or concerns, a sample collection tube, and a stamped envelope to mail the collected sample to the laboratory at no cost to the patient. All participants were asked to return the FIT within 2 months from the mailing date. Those in the control arm received no financial incentive. Those in the unconditional incentive arm received a $10 store gift card along with their FIT. Those in the conditional incentive arm received a letter with their FIT that indicated they would receive a $10 gift card if they completed and returned the enclosed FIT within 2 months. Those in the lottery incentive received a letter with their FIT indicating that if they completed and returned the enclosed FIT within 2 months they would be entered in a lottery with a 1-in-10 chance of receiving a $100 gift card. The gift card was from a national pharmacy retail chain with a local presence.

Participants who did not return a completed FIT within 3 weeks received an interactive voice response reminder call. Participants who did not complete the test within the following 3 weeks (6 weeks from initial mailing) were mailed a reminder letter. All FIT results were sent to the patient’s primary care clinician, and patients with negative results received a letter. For patients with positive results, research staff contacted the primary care clinician directly to coordinate follow-up diagnostic colonoscopy; in such cases, patients received both a telephone call and a follow-up letter. The investigators were blinded to patient data and randomization, but the research staff were not blinded as they were administering the interventions.

### Postintervention Interviews

Twelve weeks after the date of mailing FIT to patients, a random 20% subsample was selected to receive a follow-up semistructured telephone interview about their experiences completing the FIT kit and their beliefs surrounding incentives for FIT kit completion (eFigure in Supplement 2). Research staff made up to 3 attempts to contact the selected participants.

### Study Outcomes

The primary outcome of interest was the percentage of patients that completed the mailed FIT within 2 months of mailing. Secondary outcomes included the percentage of patients who returned a FIT within 6 months and number of patients receiving any CRC screening within 6 months of mailing. We also tracked the outcomes of patients with positive FIT, including receipt of colonoscopy and findings during colonoscopy. Data were obtained from the EHR. Race and ethnicity data were based on self-reported data in the EHR to evaluate response rate, as many studies have shown racial and ethnic differences in screening participation. Household income was estimated using the American Community Survey 2012-2016 5-Year Estimates data for median income by zip code of residence.

### Statistical Analysis

Based on preliminary data, it was anticipated that 1080 of 1350 participants from the automated data pull would be eligible and randomized, with 270 participants enrolled on to each of the 4 arms in a 1:1:1:1 ratio. A base return rate for the control group was estimated to be 15% based on a quality improvement pilot in this clinic population. Each intervention arm was compared with the control with a 2-sided *P* < .017 (0.05/3) as statistically significant, to account for 3 comparisons with Bonferroni correction. Thus, we anticipated 80% power to detect an absolute 11–percentage point increase in response rate. Due to concurrent CRC screening initiatives at the practice, only 897 of the 1373 participants identified by the EHR pull were found to be eligible. We report response rates as a proportion with 95% confidence intervals. Comparisons between arms were performed using the test of proportions (prtesti) between each incentive arm and the control arm for the 2-month and 6-month response rate. All analyses were performed in Stata, version 15.0 (Stata Corp LP).

## Results

### Patient Characteristics

A total of 1373 potentially eligible patients were identified through automated data extraction; 897 of these were included after medical record review and randomly allocated to the 4 study arms using a computerized random number generator (Figure 1). Among the randomized patients, 17 patients (6 in the control arm, 5 in the unconditional arm, 5 in the conditional arm, and 1 in the lottery arm) had unopened kits returned owing to incorrect mailing addresses and did not receive the intervention, although they were included in the intent-to-treat analysis. The intervention was conducted from December 2015 to February 2018, when 6-month follow-up was completed for all randomized participants.

The median age of patients was 57 years (interquartile range, 52-62 years) (Table 1). Overall, most patients included in our study were women (56%) and black (69%). Most patients had commercial insurance (54%) or Medicare (25%); 21% had Medicaid. Median household income was $31 113 (interquartile range, $26 356-$47 945).

**Table 1.** Demographic Characteristics by Group Assignment

Characteristic	Study Arm, No. (%)			
	No Incentive (n = 223)	Unconditional Incentive (n = 224)	Conditional Incentive (n = 224)	Lottery Incentive (n = 226)
Women	126 (56.5)	123 (54.9)	123 (54.9)	132 (58.4)
Age, median (IQR), y	56 (52-61)	56 (52-63)	58 (52-63)	56 (52-62)
Race				
Black	150 (67.3)	165 (73.6)	151 (67.4)	151 (66.8)
White	53 (23.8)	39 (17.4)	47 (21.0)	46 (20.4)
Asian/Pacific Islander	8 (3.6)	9 (4.0)	15 (6.7)	16 (7.1)
Other	12 (5.4)	11 (4.9)	11 (4.9)	13 (5.8)
Ethnicity				
Hispanic	8 (3.6)	5 (2.2)	3 (1.3)	6 (2.7)
Non-Hispanic	213 (95.5)	218 (97.3)	220 (98.2)	220 (97.4)
Unknown	2 (0.9)	1 (0.4)	1 (0.4)	0
Insurance type				
Commercial	120 (53.8)	119 (53.1)	119 (53.1)	123 (54.4)
Medicare	56 (25.1)	53 (23.7)	60 (26.8)	57 (25.2)
Medicaid	47 (21.1)	52 (23.2)	44 (19.6)	45 (19.9)
Self-pay	0	0	1 (0.5)	1 (0.4)
Household income, median (IQR), $^a^	30 797 (27 942-44 809)	30 797 (26 356-52 433)	30 797 (26 356-52 433)	31 113 (26 356-47 945)

Abbreviation: IQR, interquartile range.

^a^ Based on American Community Survey 2012-2016 5-Year Estimates data by zip code of residence.

### FIT Completion

**Figure 2.**
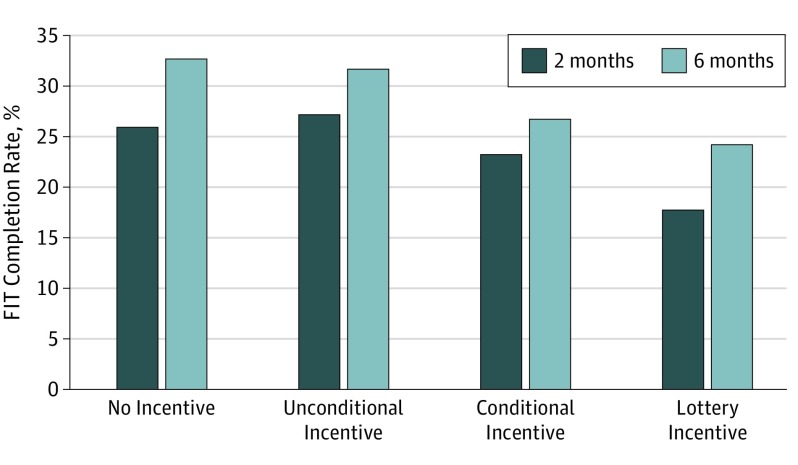
Mailed Fecal Immunochemical Test (FIT) Completion by Arm

In intent-to-treat analysis, 211 patients (23.5%) returned a FIT by mail within 2 months of receipt. The completion rate at 2 months was 26.0% (95% CI, 20.4%-32.3%) in the control group, 27.2% (95% CI, 21.5%-33.6%) in the unconditional incentive arm, 23.2% (95% CI, 17.9%-29.3%) in the conditional incentive arm, and 17.7% (95% CI, 13.0%-23.3%) in the lottery incentive arm (Table 2). None of the arms with an incentive were statistically superior to the arm without incentive. The overall FIT completion rate was 28.9% at 6 months, and there was also no significant difference by arm. The completion rate at 6 months was 32.7% (95% CI, 26.6%-39.3%) in the no incentive arm, 31.7% (95% CI, 25.7%-38.2%) in the unconditional incentive arm, 26.8% (95% CI, 21.1%-33.1%) in the conditional incentive arm, and 24.3% (95% CI, 18.9%-30.5%) in the lottery incentive arm (Figure 2). Women had a 25.8% response rate and black patients had a 22.0% response rate at 2 months, with no difference in completion rate across arms based on sex or race.

**Table 2.** Response to Outreach and Completion of FIT

FIT Kit Completion	No Incentive (n = 223)	Unconditional Incentive (n = 224)	Conditional Incentive (n = 224)	Lottery Incentive (n = 226)
Within 2 mo				
No.	58	61	52	40
% (95% CI)	26.0 (20.4-32.3)	27.2 (21.5-33.6)	23.2 (17.9-29.3)	17.7 (13.0-23.3)
*P* value (compared with no incentive)^a^	NA	.77	.49	.03
Within 6 mo				
No.	73	71	60	55
% (95% CI)	32.7 (26.6-39.3)	31.7 (25.7-38.2)	26.8 (21.1-33.1)	24.3 (18.9-30.5)
*P* value (compared with no incentive)^a^	NA	.82	.17	.05

Abbreviations: FIT, fecal immunochemical test; NA, not applicable.

^a^ *P* < .017 (0.05/3) was the threshold for statistical significance with Bonferroni correction to account for 3 pairwise comparisons.

Among all patients returning a FIT during the follow-up period, 23 (9%) had a positive result and 16 (70%) of those patients received a follow-up colonoscopy. Among the 16 who received a follow-up colonoscopy, adenomas were found in 10 (62.5%) of those patients, including 2 patients with high-grade dysplasia and 1 with a cancerous polyp.

At 6 months, 43 patients (4.8%) received screening colonoscopy outside of study outreach, including 10 (4.5%) in the control arm, 12 (5.4%) in the unconditional incentive arm, 9 (4.0%) in the conditional incentive arm, and 12 (5.3%) in the lottery incentive arm. Three participants (0.3%) received FIT (n = 2) or fecal occult blood test (n = 1) outside of study outreach.

### Postintervention Interviews

Of the 897 patients in the study, 168 (19%) were called to complete the postintervention interview, and 74 (44%) agreed to participate. When asked whether they would prefer a FIT or colonoscopy for future screening, 39 (53%) stated they would prefer to receive screening using a FIT; 23 (31%) preferred colonoscopy, and 9 (12%) had no preference. Among the 70 patients who responded, a preferred method of receiving reminders for future screenings was by letter (23 [33%]), telephone (19 [27%]), text (15 [21%]), email (14 [20%]), or via a discussion with their physician (9 [13%]). Among the 31 patients asked about incentives, respondents listed that cash (11 [35%]), check (10 [32%]), or gift card (8 [26%]) are the preferred methods for receiving incentives and that a median incentive amount of $15 (interquartile range, $0-$50) would be sufficient to compensate for the time and effort required to complete and return a FIT.

## Discussion

In this study, we found an overall mailed FIT completion rate of 23.5% at 2 months and 28.9% at 6 months. This was a substantial rate of CRC screening uptake in a population that was not up-to-date and typically received recommendation for colonoscopy in the clinic setting. In addition to a conditional incentive that offered $10 after completion of the test, we also evaluated behavioral economic incentives such as a 1-in-10 chance of winning $100 or an unconditional $10 incentive that was sent with the FIT kit. However, the different forms of financial incentives of the same expected value ($10) were not more effective than mailed FIT outreach alone in the completion rates.

There is some prior evidence that financial incentives to patients can increase CRC screening rates,^19,21^ and the design of our incentives (lottery and unconditional incentives) incorporated behavioral economic principles that have been shown to increase response in health and nonhealth contexts. Additionally, the incentives were complementary to a mailed FIT program with a high response rate associated with what has been seen in prior studies.^10,30,31^ Why were financial incentives not effective in this context?

First, the amount of the incentive may have been too small. Traditional economics shows that a larger financial incentive is associated with greater impact. That may also hold true for financial incentives to patients, as the value gained by the patient can overcome present-time bias and the perceived burden associated with completing the test.^32^ The results of our follow-up surveys suggested that a median of $15 may be necessary to account for the time and effort to participate. However, Kullgren et al^19^ showed that a smaller lottery-based incentives of $5 expected value was effective for a similar stool test in CRC screening. We chose $10 based on this prior study and what might be feasible for practices to offer for an annual screening test. While larger incentives may be more effective, they may not be scalable for organizations who would have to invest in this program every year. Future FIT screening cost-effectiveness analyses could identify a range of financial incentive threshold amounts, accounting for response rates, for evaluation in pragmatic trials.

Second, the incentives may not have been communicated effectively. The prior study by Kullgren et al^19^ showing benefit from incentives offered the stool testing kit and incentive to participants in an in-person clinic setting. In our study, the incentives were communicated by mail with no follow-up telephone call to discuss the incentive offer. While effort was made to highlight the incentives, patients may have missed or not understood the explanation. Future efforts could consider follow-up telephone calls or offering incentives in a clinical setting, but the added cost of such interventions needs to be considered.

Third, the study may not have been powered adequately to detect small differences in response rate. The study was initially powered to detect a difference of 11 percentage points, but due to concurrent clinic efforts to increase screening rates, fewer patients were included in the trial. Additionally, the response rate in the control group (26%) was higher than anticipated owing to process improvements that were incorporated into the trial such as a follow-up letter and automated telephone call. Revised power calculations indicate approximately 80% power to detect a difference of 14 percentage points in the intervention groups. Financial incentives may have a smaller effect size, but practices would need to see sufficient response to justify investing in financial incentives as compared with other outreach efforts like telephone calls or repeated mailings. For this population, our findings suggest that outreach alone was adequate without the potential cost or complexity of incentive-based designs.

Fourth, the design of the incentive may not have been salient enough to patients. Since the patient had to mail back the FIT kit and then receive the incentive at a later date, it may not have been enough to overcome present-time bias. The unconditional incentive had a higher response rate than the 2 conditional incentive arms, which is consistent with prior studies.^26,27^ The lottery incentive had the lowest response rate, which is consistent with literature showing that individuals are risk averse for gains, even in low-payoff settings.^14,33^ We also offered a gift card from a national pharmacy, which may not have been accessible or useful to the patient. Additionally, while the FIT kits were sent from the clinical practice, there may have been a situational dissonance about the incentive since it is not a typical offering from a primary care clinician. There is also the possibility that financial incentives may not work in this context to overcome inertia or the unpleasant aspect of completing the stool test.

### Strengths and Limitations

The strengths of this study include its prospective design and patient-level randomization. As a pragmatic trial conducted in close partnership with a primary care practice, we showed how FIT outreach can effectively complement in-clinic CRC screening recommendations, which is generalizable to many clinical environments. In an attempt to increase the efficiency of this approach, we relied only on mailings and not follow-up telephone calls or repeat FIT kits that may limit scalability. We also incorporate incentive design grounded in behavioral economic principles that have been shown to be effective in other settings. The practice setting also includes an urban population that was predominantly black with a median household income of approximately $40 000 per year. While these groups have had limited CRC screening rates and lower response to outreach efforts,^11,13,34,35^ we show similar response rates in this program.

This study also has some limitations. First, we focused only on FIT outreach, and there was no option for colonoscopy. However, colonoscopy is the predominant form of screening in this population, so FIT provides a complement to existing clinic-based efforts. Second, our primary study outcome followed patients for only 2 months, but a secondary outcome looked at 6-month follow-up, which was slightly higher and showed no differences across arms. Third, there was limited power to detect small differences between arms. Fourth, we examined only financial incentives in the context of mailed outreach, with no interaction on the telephone or in person.

## Conclusions

Our study showed high uptake of mailed FIT, but financial incentives of $10 equivalent value did not affect screening response rates in this population. Further efforts are needed to enhance uptake of CRC screening to make it more scalable and effective.

## Notes

Supplement 1. **Trial Protocol**

Supplement 2. **eTable.** Inclusion and Exclusion Criteria for Data Extraction **eFigure.** Postintervention Interview Guide

Supplement 3. **Data Sharing Statement**
