# Errors in Text and Conflict of Interest Disclosures

**DOI:** 10.1001/jamanetworkopen.2019.3771

**Published:** 2019-04-26

In the Original Investigation titled “Effect of Financial Incentives on Patient Use of Mailed Colorectal Cancer Screening Tests: A Randomized Clinical Trial,”^1^ published March 22, 2019, there was an error in the first sentence of the Conclusions section. It should read as follows: “Our study showed high uptake of mailed FIT, but financial incentives of $10 equivalent value did not affect screening response rates in this population.” Also, in Conflict of Interest Disclosures, Ms Saia should have reported no disclosures. This article has been corrected.^1^
