# Supplementary material for: Effect of Financial Incentives on Patient Use of Mailed Colorectal Cancer Screening Tests: A Randomized Clinical Trial
Source: JAMA Netw Open. 2019 Mar 22;2(3):e191156. doi: 10.1001/jamanetworkopen.2019.1156 (PMC6583304; doi:10.1001/jamanetworkopen.2019.1156)
Supplement: Supplement 1. — Trial Protocol [file jamanetwopen-2-e191156-s001.pdf]

Supplement

Single Center Randomized Pilot Study of Colorectal Cancer Screening Outreach (CRCSO)

This supplement provides additional information about the work. It contains the following items:

Contents

Initial Protocol 2

Final Protocol 10

Summary of Protocol Changes Modifications 18

Initial Statistical Analysis Plan 20

Final Statistical Analysis Plan 21

Summary of Statistical Analysis Plan Modifications 22

SINGLE CENTER RANDOMIZED PILOT STUDY OF COLORECTAL CANCER SCREENING OUTREACH

Principal Investigator Shivan Mehta MD, MBA, MSHP
shivan.mehta@uphs.upenn.edu

Co-Principal Investigator Chyke Doubeni, MD, MPH
chyke.doubeni@uphs.upenn.edu

IRB Protocol Number: 823388

UPCC Protocol Number: 17215

ClinicalTrials.gov Number NCT02594150

Table of Contents

1 ABSTRACT 11
2 OBJECTIVES 11
3 BACKGROUND 11
4 STUDY DESIGN 11
5 HUMAN SUBJECTS 12
6 PROCEDURES 13
7 ANALYSIS PLAN 15
8 DATA AND SAFETY MONITORING 15
9 RISK/BENEFIT ASSESSMENT 17

1. Abstract

This pilot study is a 4-arm randomized control trial assessing the effectiveness of financial incentives for mailed fecal immunochemical test (FIT) kits in patients between 50 and 75 years of age, who have received care Penn Family Care, are due for screening, and are asymptomatic for CRC. The two arms are: (1) usual care, (2) unconditional incentive, (3) conditional incentive, and (4) lottery incentive.

2. Objectives

Primary Objective is to assess the effectiveness of financial incentives to increase the response rates to mailed FIT.

Secondary Objective is to further our understanding of the impact of financial incentives on completing mailed FIT.

Primary Study Endpoint will be an increase in return rate of completed FITs between the intervention arms when compared to the control arm.

Secondary Study Endpoints n/a (the secondary objective is qualitative in nature and thus there is no quantitative endpoint).

3. Background

Colorectal cancer (CRC) is the second leading cause of cancer death in the United States (US), but CRC is potentially preventable through screening. CRC is most curable when detected at an early stage, but unfortunately, over one-half of tumors are detected at late-stage. Screening is cost-effective and has been a key contributor to the declining US CRC incidence and mortality rates over the past three decades. However, screening is underutilized in the US, particularly in some population groups such as blacks, contributing to racial/ethnic disparities in mortality for CRC.

National guidelines recommend screening in average-risk adults aged 50-75. Microsimulation studies suggest that, with high levels of adherence, screening with fecal immunochemical test (FIT) annually may be comparably effective for reducing the risk of CRC death as screening with colonoscopy every 10 years. However, such high levels of FIT adherence are nearly impossible to achieve without effective programs to overcome barriers to receipt of screening.

FIT is ideally suited for mailed outreach screening. FIT has higher sensitivity and specificity and is more acceptable because of improved collection devices and fewer required samples compared to gFOBT. FIT is specific for human hemoglobin thus eliminating dietary restrictions. FIT's performance characteristics have been studied in many settings. In a recent large US study, OC FIT-CHEK's specificity was 94.9% and its sensitivity for detecting advanced adenomas and cancers was 23.8% and 73.8%, respectively.

In this study, we will be using population-based outreach screening to overcome barriers in CRC screening uptake. Mailed FIT outreach has been used to successfully increase CRC screening uptake in many settings, including in Kaiser Permanente Northern California to increase their screening rates to over 80%. Yet, these types of organized programs are limited by response rates to mailed FIT and the costs of annual outreach. There is an opportunity to leverage an organized approach in a different health system but also evaluate new ways to increase response rate. By evaluating the effectiveness of offering financial incentives paired with mailed outreach screening, we will enhance the public health capacity, and efficiency, to increase CRC screening uptake and reduce preventable death from this disease, particularly in underserved populations.

We will also test behavioral economic engagement incentives. Behavioral economics applies principles from both psychology and economics to human health behavioral practices. People do not dispassionately consider the costs and benefits of the choices they make, but tend to place a disproportionately high value on immediate costs and benefits of their actions. This *present-time bias* typically works against health behaviors, since the benefits are typically delayed, while the cost is immediate. There is a significant and immediate non-monetary cost to getting screened that includes the unpleasant nature of and opportunity cost in performing the test, or fear of the results. Financial incentives have been effective in producing desired behavior actions in a variety of clinical contexts, and have been studied in breast and cervical cancer screening, but evidence for use in CRC screening is limited.

4 Study Design

Design

A single center 4-arm randomized pilot study to assess if the rate of completion of FIT is increased by the addition of financial incentives. Potentially eligible subjects will be identified via a data abstraction by Penn Data Store. We will randomly select patients and conduct an electronic medical record (EMR) review using PennChart to confirm eligibility. We will then randomize 1,080 of remaining eligible patients to one of four arms during the intervention phase.

1. Arm 1: mailed FIT kit (usual care)
2. Arm 2: mailed FIT kit + $10 gift card included in mailing (reciprocity)
3. Arm 3: mailed FIT kit + guarantee of receiving a $10 gift card if kit is returned within two months (loss aversion)
4. Arm 4: mailed FIT kit + entry in a 1/10 chance lottery to receive $100 gift card if kit is returned within two months (lottery)

All 1,080 subjects will receive the mailed FIT kit. Subjects whose FIT results are not recorded in PennChart will receive a follow-up phone call via Interactive Voice Response (IVR) three weeks after the kit was mailed to the subject. Subjects whose FIT results are not recorded in PennChart will receive a follow-up mailed reminder six weeks after the kit was mailed to the subject. Subjects will receive follow-up regardless of the arm to which they are randomized.

A random subsample of 200 subjects will be called twelve weeks after the kit was mailed to the subject to complete a questionnaire. Verbal consent will be obtained from this subsample. This phone call will occur after the intervention and follow-up phases are completed. Analysis will occur after the subsample questionnaire phase is completed.

Duration

Subject duration: We anticipate it taking the subject no more than 15 minutes to prepare, complete, and mail the FIT.

Study duration: We anticipate participant enrollment to occur in batches on a rolling basis, such that each of nine batches will include two months of chart review and sending of FIT kits. Given this in conjunction with a two-month wait period (after the last kit is mailed), and a three month analysis period, we anticipate this pilot project to last 23 months.

5. Human Subjects

Resources Necessary for Human Research Protections
Safety will be monitored on an ongoing basis by the PI and the study team. The PI or designee will review the study charts to evaluate events at each subject interaction to ensure the grade, relationship to the study procedure, expectedness and the course of action for each subject is documented. The PI or Sub-investigator is ultimately responsible for assigning grade and attribution.

Subjects Enrolled by Penn Researchers 1080

Subjects Enrolled by Collaborating Researchers 0

Accrual
Patients will not be actively recruited for this protocol. We will submit a data query of Penn Family Care patients from the Penn Data Store based on an electronic EMR algorithm that determines guideline-concordant colorectal cancer screening within PennChart. We will randomize 1,080 subjects. We will randomly select 200 patients from the 1,080 subject population with whom we will contact to conduct the questionnaire.

Key Inclusion Criteria
1. Between 50 to 75 years old
2. Has received care Penn Family Care
3. Due for screening
4. Asymptomatic for CRC

Key Exclusion Criteria
1. Has had prior colonoscopy within 10 years, sigmoidoscopy within 5 years, and FOBT/FIT within twelve months of the chart review (We will exclude patients who self-report undergoing any of the above procedures)
2. Has a history of CRC
3. Has a history of other GI cancer
4. Has history of confirmed Inflammatory Bowel Disease (IBD) e.g. Crohn’s disease, ulcerative colitis. Irritable bowel syndrome does not exclude patients
5. Has history of colitis other than Crohn’s disease or ulcerative colitis
6. Has had a colectomy
7. Has a relative that has been diagnosed with CRC
8. Has been diagnosed with Lynch Syndrome (i.e. HNPCC)
9. Has been diagnosed with Familial Adenomatous Polyposis (FAP)
10. Has anemia (iron-deficient)
11. Has history of lower GI bleeding
12. Has metastatic (Stage IV) blood or solid tumor cancer
13. Has end stage renal disease
14. Has any other condition that, in the opinion of the investigator, excludes the patient from participating in this study

Vulnerable Populations

We are not targeting any vulnerable populations.

Subject Compensation
No stipends will be provided. There is the potential of receiving financial incentives in the form of gift cards. In Arm 2, subjects will receive a $10 gift card regardless of whether they complete FIT. In Arm 3, subjects will receive a $10 gift card if the completed FIT is returned within two months. In Arm 4, subjects will be entered in a 1/10 chance lottery to receive $100 gift card if the completed FIT is returned within two months.

Consent
A waiver of authorization of HIPAA and waiver of consent are being sought in accordance with HHS CFR 45.46.116(d) based on the following:

- **The research involves no more than minimal risk to the subjects**: The FIT is an FDA approved, clinically available, and utilized test used to screen for colorectal cancer. Penn Family Care is already mailing out FIT kits as part of clinical care to increase screening rates. The only research related activity is the randomization of subjects to different financial incentives. Our main study objective is to assess the effectiveness of financial incentives to increase the response rates to mailed FIT. Since this study is promoting the standard of care for CRC screening, we believe waiver of consent is appropriate in this scenario and has been used in other mailed FIT and behavioral economic studies.
- **The waiver or alteration will not adversely affect the rights and welfare of the subjects**: subjects' rights and welfare will be not adversely affected by the waiver of authorization and consent. All subjects will receive a mailed FIT. Arm 1 will not receive a financial incentive (which is current standard care), Arm 2 will receive a $10 financial incentive, and Arms 3 and 4 *may* receive a financial incentive. We are not withholding any financial incentives that these patients would otherwise receive as part of clinical care. Not knowing the financial incentives are being offered for the purposes of research does not adversely affect the rights and welfare of the subjects, as their opportunity to engage in CRC screening is the same regardless of study arm. We believe it is appropriate to not obtain waiver of consent because learning the impact of financial incentives on CRC screening has a sufficient potential social value (i.e. significantly increasing the rate of CRC screening and thus decreasing the rate of CRC mortality related to nonuse of screening).
- **The research could not practicably be carried out without the waiver or alteration**: simply waiving *documentation* of consent (and thus including a consent letter that does not require a subject signature) is not sufficient in this scenario. We believe that including a consent letter in the mailings could potentially bias subjects to not participate in a clinically available and indicated screening test, and it could alter their participation. Obtaining waiver of consent would allow us to avoid the potential selection/volunteer bias for inclusion of patients particularly interested in screening that can occur when consent is required. Since our main objective is to understand the potential influence a financial incentive has on subject behavior, we believe that obtaining consent would compromise our primary objective. If this were to be scaled to routine clinical care, this type of consent would not typically be required.
- **the subjects will be provided with additional pertinent information after participation**: if the opportunity arises, we will provide additional pertinent information about CRC screening with the subject after participation. However, we are requesting waiver of debriefing since we believe that debriefing might be confusing and potentially harmful to subjects if they are upset about being deceived and no longer engage in CRC screening or engaging in primary care visits. Since the deception research is only in regards to financial incentives and not in any way related to CRC screening, the potential impact of debriefing on future CRC screening supports our request to waive debriefing.

6. Procedures

Screening Phase
Patients will not be actively recruited for this protocol. We will submit a data request of Penn Family Care patients from the Penn Data Store based on an electronic EMR algorithm that determines guideline-concordant colorectal cancer screening within PennChart. We estimate 1,350 screen-eligible patients will be identified through this EMR query. We will then conduct an EMR review to confirm eligibility. Through the PFC quality improvement initiative, we have found the accuracy rate of the EMR algorithm to be approximately 80%. As such, we anticipate 1,080 subjects to be eligible, all of whom we will randomize to one of the four arms.

Study Intervention Phase
FIT is an immunological stool-based screening test for colorectal cancer. It tests for the presence of human hemoglobin in feces, which increases sensitivity and specificity while decreasing false positives and false negatives. There are no dietary restrictions and requires no direct fecal handling. This is a laboratory test that is used in clinical care and it is a commercially available device. The FIT brand that will be used for this protocol is the OC-Auto® Personal Use Kit manufactured by Polymedco, Inc. FIT is reimbursed by all major insurance companies, Medicare, and Medicaid. .

Eligible subjects will be randomized to one of four arms during the intervention phase:

1. Arm 1: mailed FIT kit (usual care)
2. Arm 2: mailed FIT kit + $10 gift card included in mailing (unconditional incentive)
3. Arm 3: mailed FIT kit + guarantee of receiving a $10 gift card if kit is returned within two months (conditional incentive)
4. Arm 4: mailed FIT kit + entry in a 1/10 chance lottery to receive $100 gift card if kit is returned within two months (lottery incentive)

270 subjects will be randomized to each arm. The investigator and program manager will be blinded to randomization; the research coordinator and assistants will not be blinded. FIT kits will be mailed to subjects over approximately 18 months. Each kit will include a tube in which to deposit the stool sample, directions on how to collect and mail the sample, a letter about CRC screening, a Labcorp requisition form, and a pre-paid return envelope. All subjects will be asked to return the completed kit within two months of receiving the FIT kit.

In addition to the FIT, subjects randomized to Arm 2 will also receive a $10 gift card and a note explaining that this gift card is a token of our appreciation for completing the kit. Subjects randomized to Arm 3 will receive a note stating that they will receive a $10 gift card if they return their completed FIT within two months of receiving the FIT kit. Subjects randomized to Arm 4 will receive a note stating that they will be entered in a 1/10 chance lottery to receive a $100 gift card if they return their completed FIT within two months of receiving the FIT kit.

Follow Up Phase

Follow-up is the same for all subjects regardless of the arm to which subjects are randomized. Subjects who have not returned kit 3 weeks (±3 days) after FIT mailing will be contacted using Interactive Voice Response (IVR) to be reminded to complete the kit. Subjects who have not returned kit at least 6 weeks (±3 days) after FIT mailing (and 3 weeks after IVR phone call reminder) will receive a reminder letter in the mail reminding them to complete the kit. We will also conduct a 6-month follow-up via automated chart audit to see if CRC screening was received by participants.

When a negative result is reported, the primary care physician will be notified via PennChart. If the subject utilizes MyPennMedicine, he/she will be sent a message via MyPennMedicine to communicate the negative results.

When a positive result is reported, the study team will notify the primary care physician and request a colonoscopy referral to the GI department, obtain an order number for a colonoscopy, and then schedule the colonoscopy with the subject and the GI department over the phone.

Sub-Sample Questionnaire Phase

A random subsample of 200 subjects will be selected to complete a questionnaire 9 weeks (±3 days) after they received the mailing. Verbal consent will be obtained from this subsample before the questionnaire is administered. Through this questionnaire, the subjects will be asked to confirm their eligibility (e.g. that they had not had CRC screening within the United States Preventive Services Task Force (USPSTF) CRC screening guidelines) and provide additional demographic and socioeconomic status information so that we can better understand what populations, if any, may be more likely impacted by the financial incentives when engaging in CRC screening. We anticipate these questionnaires to take 15-30 minutes to complete over the phone. The research staff will make no more than three attempts to speak directly with the subject. Based on the Penn Family Care quality improvement initiative where we reached about 50% of patients via phone call, we anticipate reaching approximately 100 subjects to complete this sub-sample questionnaire.

Randomization

Using Stata and Excel, subjects will be assigned randomly assigned Study ID numbers and then randomized using a computer-generated randomization algorithm. The research coordinator will record the randomization assignments on a master list which will be maintained by the research coordinator on a password protected computer in a locked office. The research coordinator and research assistants will assemble the mailings based on this master list.

Subject Withdrawal

Subjects will be withdrawn from this study if they contact the study team and request to be withdrawn from this study. Withdrawal will not affect subject safety or the subject's care at Penn Family Care.

Subjects may withdraw from the study at any time without impact to their care. They may also be discontinued from the study at the discretion of the Investigator (e.g. subject self-reports previous CRC screening that meets the USPSTF CRC screening guidelines, subject contacts the study team and requests to be withdrawn from the study, etc.). The Investigator may also withdraw subjects to protect the subject for reasons of safety or for administrative reasons. It will be documented whether or not each subject completes the clinical study. No protected health information from PennChart

will be maintained on behalf of this study. De-identified demographic information will be maintained for analysis purposes. The below table depicts the timeline of study enrollment described above.

	Pre-screening	Baseline<br>Day 0	FU to Mailing<br>Wk 3 (±3 days)	FU to Mailing<br>Wk 6 (±3 days)	FU to Mailing<br>Wk 9 (±3 days)
EMR review	X				
Randomization	X				
Mail FIT		X			
IVR			X		
Mailed reminder				X	
Questionnaire (subsample)					X

7. Analysis Plan

Power and Sample Size
Through the PFC quality improvement initiative, we have found the accuracy rate of the EMR algorithm to be approximately 80%. As such, we anticipate 1,080 of 1,350 subjects will be eligible with 270 subjects enrolled on to each of the four arms in a 1:1:1:1 ratio. We estimate a base return rate for controls to be 15% and a base return rate for the intervention arms to be 25%. We will compare the intervention arms against the control arms but we will not compare one intervention against another intervention. We have 80% power to detect an absolute 10% increase in response rate using a two-sided Type 1 error rate of 0.05.

Data Analysis
We will conduct a chi-square analysis using statistical software Stata 12 to compare each intervention arm to the control arm. A second chi-square analysis will be performed by blinded members of the research team to compare response rates of the study participants and patients receiving FIT as standard clinical care. Analysis will be conducted by the research team twelve weeks after the last subsample questionnaire is completed.

Subject Population(s) for Analysis
The population that will be included in the primary study analysis will include subjects who were randomized into the study and who completed FIT as an intention to treat analysis. Two populations will be included in the secondary analysis. The first will include any subjects randomly selected to receive a subsample questionnaire and who completed the questionnaire. The second will include patients who currently receive FIT as standard of care in the Penn Family Care Clinic. In order to provide a comparison group, retrospective chart review will be performed on this group in order to compare response rates and demographics between study participants and the eligible clinic population. The data collected on these patients will be identical to the data collected on study participants (i.e. demographics, inclusion criteria, exclusion criteria, response rates).

8. Data and Safety Monitoring

Protected Health Information/Data Protection
We will abstract the following variables from PennChart.

- Name
- Address
- Birth date
- Date of most recent CRC screening (e.g. colonoscopy, sigmoidoscopy, FOBT/FIT)
- Medical and family history
- Phone number
- Medical record number

Recording of Adverse Events
At each contact with the subject, the investigator will seek information on adverse events by specific questioning. Information on all adverse events will be recorded immediately in the source document, and also in the appropriate adverse event module of the case report form (CRF). All adverse events occurring during the study period will be recorded. The clinical course of each event will be followed until resolution, stabilization, or until it has been determined that the study intervention or participation is not the cause. Serious adverse events that are still ongoing at the end of the study period will be followed up to determine the final outcome. Any serious adverse event that occurs after the study period and is considered to be possibly related to the study intervention or study participation will be recorded and reported immediately.

The relationship of each adverse event to the study procedures will be characterized by the PI and will be classified as either definitely related, probably related, unlikely to be related, or unrelated.

Reporting of Adverse Events, Adverse Device Effects and Unanticipated Problems
The PI and study team will conform to the adverse event reporting timelines, formats and requirements of the IRB and DSMC. If a narrative report is submitted, the following information will be provided to all reviewing entities:

Study identifier	Current status
Study Center	Whether study intervention was discontinued
Subject number	The reason why the event is classified as serious
A description of the event	Investigator assessment of the association between the event and study intervention
Date of onset	

Any other events will be recorded and reported in accordance with institutional and federal policies.

If an SAE has not resolved at the time of the initial report and new information arises that changes the investigator's assessment of the event, a follow-up report including all relevant new or reassessed information will be submitted to the IRB and DSMC. The PI will be responsible for ensuring that all SAE are followed until either resolved or stable.

Investigator Reporting: Notifying the Penn IRB
The investigator will submit reports to the IRB within ten working days (with one exception) of events that meet the definition of an unanticipated problem involving risks to subjects or others. Exception: If the adverse event involved a death and indicates that participants or others are at increased risk of harm, the investigator will submit a report to the IRB within three days.

The investigator will submit reports to the IRDSMC in accordance to their current reporting requirements:
All Grade 3 or higher events (AE or SAE) within five business days of knowledge.
All unexpected deaths within 24 hours of knowledge.
All others deaths within 30 days of knowledge. Deaths of subjects off-study for greater than 30 days from the last study treatment/intervention are not reportable.

Unblinding Procedures
Only the PI, co-investigator, and project manager will be blinded to what financial incentive subjects are randomized. If unblinding of members of the research team becomes necessary, we will document the following information in REDCap: why unblinding was necessary, date of approval to unblind from the IRB and DSMC, when unblinding occurred, and how unblinding occurred.

Confidentiality
Information about study subjects will be kept confidential and managed according to the requirements of the Health Insurance Portability and Accountability Act of 1996 (HIPAA). Please refer to our request to waive HIPAA authorization.

Data Collection and Management
Source documents are maintained in PennChart. No source documents will be printed or maintained in paper form at the study site. Data from PennChart will be recorded in Case Report Forms (CRFs) developed within Penn Medicine's REDCap system. The investigator and study team (which includes the project manager, research coordinator, and research assistants) will have access to PHI within PennChart and REDCap. We will label all PHI within REDCap as identifiable information so that de-identified exports are possible. All reports that include identifiable information will be stored on the Department of Family Medicine's secure drive, maintained behind the UPHS firewall. Once data analysis and manuscripts have been published, the databases will be removed from REDCap and the data will be de-identified on the secure drive. This de-identified dataset will be stored for up to five years after analysis is complete and manuscripts have been published

Records Retention
Once analysis is completed and any manuscripts are published, we will retain personally identifiable information no longer than seven years in accordance with government regulations, applicable policies, and institutional requirements.

Study Monitoring Plan
The Principal Investigator will monitor this study with the research team on a bi-weekly basis. He will review the study progress through regular electronic and in person communication.

Auditing and Inspecting

At the discretion of the DSMC, this protocol will be audited approximately 12-18 months from the first subject enrolled and approximately every other year thereafter for the duration of the study. The Principal Investigator will be notified in advance of the selection of their protocol for review and the cases that have been randomly selected. Three randomly selected subjects or 10% of the total accrual, whichever is higher, will be audited. The committee may alter the frequency of re-monitoring based on the audit findings and degree of deficiencies.

The Principal Investigator will permit study-related monitoring, audits, and inspections by the DSMC and/or IRB, government regulatory bodies, and University compliance and quality assurance groups of all study related documents (e.g. source documents, regulatory documents, data collection instruments, study data etc.). The Principal Investigator will ensure the capability for inspections of applicable study-related facilities. As the Principal Investigator of this study, I accept the potential of inspection by government regulatory authorities and applicable University compliance and quality assurance offices.

9. Risk/Benefit Assessment

Risks
The risks associated with this study are no more than minimal. There is the potential risk of breach of confidentiality. We will minimize this risk by using de-identified information whenever possible and by maintaining all identifiable information on a secure drive and/or in a HIPAA-compliant system (e.g. REDCap). There is also the risk of psychological harm associated with being screened for cancer. We will minimize this risk by communicating the results of the screening test to the subject in a timely fashion and facilitating the scheduling of diagnostic testing if the screening test is positive.

Benefits
If a participant completes and returns the FIT, which is standard clinical care, the subjects will potentially benefit from participation by increasing the chances of identifying colorectal cancer at an early stage. Information learned from this study may benefit society through a better understanding of how to effectively increase the return rate of mailed CRC screening which could in turn increase the rate of CRC screening and reduce the rate of CRC mortality.

Risk Benefit Assessment
The risks associated with this study are no more than minimal. Better knowledge of how to increase mailed screening could potentially address one of the major barriers of accessing care, (i.e. having patients come in for clinical office visits). Additionally, FIT is less invasive than colonoscopy and, according to the USPSTF, considered equally effective if conducted once a year (as opposed to having a colonoscopy once every ten years). For these reasons and those outlined in the above benefits section, the Principal Investigator believes that the risks of participating in the study are outweighed by the potential benefits of participating in the study.

Final Protocol

****New changes from initial protocol notated in bold, parts removed from initial protocol notated in strikethrough**

SINGLE CENTER RANDOMIZED PILOT STUDY OF COLORECTAL CANCER SCREENING OUTREACH

**Principal Investigator**	Shivan Mehta MD, MBA, MSHP<br>shivan.mehta@uphs.upenn.edu
**Co-Principal Investigator**	Chyke Doubeni, MD, MPH<br>chyke.doubeni@uphs.upenn.edu

**IRB Protocol Number:**	823388
**UPCC Protocol Number:**	17215
**ClinicalTrials.gov Number**	NCT02594150

Table of Contents

1 ABSTRACT 11
2 OBJECTIVES 11
3 BACKGROUND 11
4 STUDY DESIGN 11
5 HUMAN SUBJECTS 12
6 PROCEDURES 13
7 ANALYSIS PLAN 15
8 DATA AND SAFETY MONITORING 15
9 RISK/BENEFIT ASSESSMENT 17

1. Abstract

This pilot study is a 4-arm randomized control trial assessing the effectiveness of financial incentives for mailed fecal immunochemical test (FIT) kits in patients between 50 and 75 years of age, who have received care Penn Family Care, are due for screening, and are asymptomatic for CRC. The two arms are: (1) usual care, (2) unconditional incentive, (3) conditional incentive, and (4) lottery incentive.

2. Objectives

Primary Objective is to assess the effectiveness of financial incentives to increase the response rates to mailed FIT.

Secondary Objective is to further our understanding of the impact of financial incentives on completing mailed FIT.

Primary Study Endpoint ~~will be an increase in~~ **is the** return rate of completed FITs **within 2 months of outreach** ~~between the intervention arms when compared to the control arm~~.

Secondary Study Endpoints ~~n/a (the secondary objective is qualitative in nature and thus there is no quantitative endpoint)~~ **include the return rate of completed FITs within 6 months of outreach, the percentage of FIT results that are positive, and the percentage of those positive subjects that receive diagnostic colonoscopy. We will look at rates of participation in FIT or colonoscopy that occur outside the study setting during the study period as an exploratory outcome. We will also evaluate the results of the qualitative survey.**

3. Background

Colorectal cancer (CRC) is the second leading cause of cancer death in the United States (US), but CRC is potentially preventable through screening. CRC is most curable when detected at an early stage, but unfortunately, over one-half of tumors are detected at late-stage. Screening is cost-effective and has been a key contributor to the declining US CRC incidence and mortality rates over the past three decades. However, screening is underutilized in the US, particularly in some population groups such as blacks, contributing to racial/ethnic disparities in mortality for CRC.

National guidelines recommend screening in average-risk adults aged 50-75. Microsimulation studies suggest that, with high levels of adherence, screening with fecal immunochemical test (FIT) annually may be comparably effective for reducing the risk of CRC death as screening with colonoscopy every 10 years. However, such high levels of FIT adherence are nearly impossible to achieve without effective programs to overcome barriers to receipt of screening.

FIT is ideally suited for mailed outreach screening. FIT has higher sensitivity and specificity and is more acceptable because of improved collection devices and fewer required samples compared to gFOBT. FIT is specific for human hemoglobin thus eliminating dietary restrictions. FIT's performance characteristics have been studied in many settings. In a recent large US study, OC FIT-CHEK's specificity was 94.9% and its sensitivity for detecting advanced adenomas and cancers was 23.8% and 73.8%, respectively.

In this study, we will be using population-based outreach screening to overcome barriers in CRC screening uptake. Mailed FIT outreach has been used to successfully increase CRC screening uptake in many settings, including in Kaiser Permanente Northern California to increase their screening rates to over 80%. Yet, these types of organized programs are limited by response rates to mailed FIT and the costs of annual outreach. There is an opportunity to leverage an organized approach in a different health system but also evaluate new ways to increase response rate. By evaluating the effectiveness of offering financial incentives paired with mailed outreach screening, we will enhance the public health capacity, and efficiency, to increase CRC screening uptake and reduce preventable death from this disease, particularly in underserved populations.

We will also test behavioral economic engagement incentives. Behavioral economics applies principles from both psychology and economics to human health behavioral practices. People do not dispassionately consider the costs and benefits of the choices they make, but tend to place a disproportionately high value on immediate costs and benefits of their actions. This *present-time bias* typically works against health behaviors, since the benefits are typically delayed, while the cost is immediate. There is a significant and immediate non-monetary cost to getting screened that includes the unpleasant nature of and opportunity cost in performing the test, or fear of the results. Financial incentives have been effective in producing desired behavior actions in a variety of clinical contexts, and have been studied in breast and cervical cancer screening, but evidence for use in CRC screening is limited.

4 Study Design

Design

A single center 4-arm randomized pilot study to assess if the rate of completion of FIT is increased by the addition of financial incentives. Potentially eligible subjects will be identified via a data abstraction by Penn Data Store. We will randomly select patients and conduct an electronic medical record (EMR) review using PennChart to confirm eligibility. We will then randomize 1,080 of remaining eligible patients to one of four arms during the intervention phase.

5. Arm 1: mailed FIT kit ~~(usual care)~~ **(control)**

6. Arm 2: mailed FIT kit + $10 gift card included in mailing (~~reciprocity~~) (**unconditional incentive**)
7. Arm 3: mailed FIT kit + guarantee of receiving a $10 gift card if kit is returned within two months ~~(loss aversion)~~ **(conditional incentive)**
8. Arm 4: mailed FIT kit + entry in a 1/10 chance lottery to receive $100 gift card if kit is returned within two months (lottery **incentive**)

All 1,080 subjects will receive the mailed FIT kit. Subjects whose FIT results are not recorded in PennChart will receive a follow-up phone call via Interactive Voice Response (IVR) three weeks after the kit was mailed to the subject. Subjects whose FIT results are not recorded in PennChart will receive a follow-up mailed reminder six weeks after the kit was mailed to the subject. Subjects will receive follow-up regardless of the arm to which they are randomized.

A random subsample of 200 subjects will be called twelve weeks after the kit was mailed to the subject to complete a questionnaire. Verbal consent will be obtained from this subsample. This phone call will occur after the intervention and follow-up phases are completed. Analysis will occur after the subsample questionnaire phase is completed.

Duration

Subject duration: We anticipate it taking the subject no more than 15 minutes to prepare, complete, and mail the FIT.

Study duration: We anticipate participant enrollment to occur in batches on a rolling basis, such that each of nine batches will include two months of chart review and sending of FIT kits. Given this in conjunction with a two-month wait period (after the last kit is mailed), and a three month analysis period, we anticipate this pilot project to last 23 months.

5. Human Subjects

Resources Necessary for Human Research Protections
Safety will be monitored on an ongoing basis by the PI and the study team. The PI or designee will review the study charts to evaluate events at each subject interaction to ensure the grade, relationship to the study procedure, expectedness and the course of action for each subject is documented. The PI or Sub-investigator is ultimately responsible for assigning grade and attribution.

Subjects Enrolled by Penn Researchers 1080

Subjects Enrolled by Collaborating Researchers 0

Accrual
Patients will not be actively recruited for this protocol. We will submit a data query of Penn Family Care patients from the Penn Data Store based on an electronic EMR algorithm that determines guideline-concordant colorectal cancer screening within PennChart. We will randomize 1,080 subjects. We will randomly select 200 patients from the 1,080 subject population with whom we will contact to conduct the questionnaire.

Key Inclusion Criteria
5. Between 50 to 75 years old
6. Has ~~received care~~ **had at least two visits** at Penn Family Care **within the past two years**
7. Due for screening
8. Asymptomatic for CRC
9. **Zip code listed in PennChart as part of the subjects' address is within the Philadelphia-Wilmington-Camden Metropolitan Statistical Area**
10. **Have a primary care provider in Penn Family Care**

Key Exclusion Criteria
15. Has had prior colonoscopy within 10 years, sigmoidoscopy within 5 years, and FOBT/FIT within twelve months of the chart review (We will exclude patients who self-report undergoing any of the above procedures)
16. Has a history of CRC
17. Has a history of other GI cancer
18. Has history of confirmed Inflammatory Bowel Disease (IBD) e.g. Crohn's disease, ulcerative colitis. Irritable bowel syndrome does not exclude patients
19. Has history of colitis other than Crohn's disease or ulcerative colitis
20. Has had a colectomy
21. Has a relative that has been diagnosed with CRC
22. Has been diagnosed with Lynch Syndrome (i.e. HNPCC)

23. Has been diagnosed with Familial Adenomatous Polyposis (FAP)
24. Has anemia (iron-deficient)
25. Has history of lower GI bleeding
26. Has metastatic (Stage IV) blood or solid tumor cancer
27. Has end stage renal disease
28. **Has had congestive heart failure**
29. **Has dementia**
30. **Has cirrhosis or end stage liver disease**
31. Has any other condition that, in the opinion of the investigator, excludes the patient from participating in this study

Vulnerable Populations
We are not targeting any vulnerable populations.

Subject Compensation
No stipends will be provided. There is the potential of receiving financial incentives in the form of gift cards. In Arm 2, subjects will receive a $10 gift card regardless of whether they complete FIT. In Arm 3, subjects will receive a $10 gift card if the completed FIT is returned within two months. In Arm 4, subjects will be entered in a 1/10 chance lottery to receive $100 gift card if the completed FIT is returned within two months.

Consent
A waiver of authorization of HIPAA and waiver of consent are being sought in accordance with HHS CFR 45.46.116(d) based on the following:

- **The research involves no more than minimal risk to the subjects**: The FIT is an FDA approved, clinically available, and utilized test used to screen for colorectal cancer. Penn Family Care is already mailing out FIT kits as part of clinical care to increase screening rates. The only research related activity is the randomization of subjects to different financial incentives. Our main study objective is to assess the effectiveness of financial incentives to increase the response rates to mailed FIT. Since this study is promoting the standard of care for CRC screening, we believe waiver of consent is appropriate in this scenario and has been used in other mailed FIT and behavioral economic studies.
- **The waiver or alteration will not adversely affect the rights and welfare of the subjects**: subjects' rights and welfare will be not adversely affected by the waiver of authorization and consent. All subjects will receive a mailed FIT. Arm 1 will not receive a financial incentive (which is current standard care), Arm 2 will receive a $10 financial incentive, and Arms 3 and 4 *may* receive a financial incentive. We are not withholding any financial incentives that these patients would otherwise receive as part of clinical care. Not knowing the financial incentives are being offered for the purposes of research does not adversely affect the rights and welfare of the subjects, as their opportunity to engage in CRC screening is the same regardless of study arm. We believe it is appropriate to not obtain waiver of consent because learning the impact of financial incentives on CRC screening has a sufficient potential social value (i.e. significantly increasing the rate of CRC screening and thus decreasing the rate of CRC mortality related to nonuse of screening).
- **The research could not practicably be carried out without the waiver or alteration**: simply waiving *documentation* of consent (and thus including a consent letter that does not require a subject signature) is not sufficient in this scenario. We believe that including a consent letter in the mailings could potentially bias subjects to not participate in a clinically available and indicated screening test, and it could alter their participation. Obtaining waiver of consent would allow us to avoid the potential selection/volunteer bias for inclusion of patients particularly interested in screening that can occur when consent is required. Since our main objective is to understand the potential influence a financial incentive has on subject behavior, we believe that obtaining consent would compromise our primary objective. If this were to be scaled to routine clinical care, this type of consent would not typically be required.
- **the subjects will be provided with additional pertinent information after participation**: if the opportunity arises, we will provide additional pertinent information about CRC screening with the subject after participation. However, we are requesting waiver of debriefing since we believe that debriefing might be confusing and potentially harmful to subjects if they are upset about being deceived and no longer engage in CRC screening or engaging in primary care visits. Since the deception research is only in regards to financial incentives and not in any way related to CRC screening, the potential impact of debriefing on future CRC screening supports our request to waive debriefing.

6. Procedures

Screening Phase
Patients will not be actively recruited for this protocol. We will submit a data request of Penn Family Care patients from the Penn Data Store based on an electronic EMR algorithm that determines guideline-concordant colorectal cancer screening

within PennChart. We estimate 1,350 screen-eligible patients will be identified through this EMR query. We will then conduct an EMR review to confirm eligibility. Through the PFC quality improvement initiative, we have found the accuracy rate of the EMR algorithm to be approximately 80%. As such, we anticipate 1,080 subjects to be eligible, all of whom we will randomize to one of the four arms.

Study Intervention Phase
FIT is an immunological stool-based screening test for colorectal cancer. It tests for the presence of human hemoglobin in feces, which increases sensitivity and specificity while decreasing false positives and false negatives. There are no dietary restrictions and requires no direct fecal handling. This is a laboratory test that is used in clinical care and it is a commercially available device. The FIT brand that will be used for this protocol is the OC-Auto® Personal Use Kit manufactured by Polymedco, Inc. FIT is reimbursed by all major insurance companies, Medicare, and Medicaid. .

Eligible subjects will be randomized to one of four arms during the intervention phase:

5. Arm 1: mailed FIT kit ~~(usual care)~~ **(control)**
6. Arm 2: mailed FIT kit + $10 gift card included in mailing (unconditional incentive)
7. Arm 3: mailed FIT kit + guarantee of receiving a $10 gift card if kit is returned within two months (conditional incentive)
8. Arm 4: mailed FIT kit + entry in a 1/10 chance lottery to receive $100 gift card if kit is returned within two months (lottery incentive)

270 subjects will be randomized to each arm. The investigator and program manager will be blinded to randomization; the research coordinator and assistants will not be blinded. FIT kits will be mailed to subjects over approximately 18 months. Each kit will include a tube in which to deposit the stool sample, directions on how to collect and mail the sample, a letter about CRC screening, a Labcorp requisition form, and a pre-paid return envelope. All subjects will be asked to return the completed kit within two months of receiving the FIT kit.

In addition to the FIT, subjects randomized to Arm 2 will also receive a $10 gift card and a note explaining that this gift card is a token of our appreciation for completing the kit. Subjects randomized to Arm 3 will receive a note stating that they will receive a $10 gift card if they return their completed FIT within two months of receiving the FIT kit. Subjects randomized to Arm 4 will receive a note stating that they will be entered in a 1/10 chance lottery to receive a $100 gift card if they return their completed FIT within two months of receiving the FIT kit.

Follow Up Phase
Follow-up is the same for all subjects regardless of the arm to which subjects are randomized. Subjects who have not returned kit 3 weeks (±3 days) after FIT mailing will be contacted using Interactive Voice Response (IVR) to be reminded to complete the kit. Subjects who have not returned kit at least 6 weeks (±3 days) after FIT mailing (and 3 weeks after IVR phone call reminder) will receive a reminder letter in the mail reminding them to complete the kit. We will also conduct a 6-month follow-up via automated chart audit to see if CRC screening was received by participants.

When a negative result is reported, the primary care physician will be notified via PennChart. If the subject utilizes MyPennMedicine, he/she will be sent a message via MyPennMedicine to communicate the negative results. **If the subject does not utilize MyPennMedicine, he/she will receive a letter informing him/her about the negative results.**

When a positive result is reported, the study team will notify the primary care physician and request a colonoscopy referral to the GI department, obtain an order number for a colonoscopy, and then schedule the colonoscopy with the subject and the GI department over the phone.

Sub-Sample Questionnaire Phase
A random subsample of 200 subjects will be selected to complete a questionnaire 9 weeks (±3 days) after they received the mailing. Verbal consent will be obtained from this subsample before the questionnaire is administered. Through this questionnaire, the subjects will be asked to confirm their eligibility (e.g. that they had not had CRC screening within the United States Preventive Services Task Force (USPSTF) CRC screening guidelines) and provide additional demographic and socioeconomic status information so that we can better understand what populations, if any, may be more likely impacted by the financial incentives when engaging in CRC screening. We anticipate these questionnaires to take 15-30 minutes to complete over the phone. The research staff will make no more than three attempts to speak directly with the subject. Based on the Penn Family Care quality improvement initiative where we reached about 50% of patients via phone call, we anticipate reaching approximately 100 subjects to complete this sub-sample questionnaire.

Randomization
Using Stata and Excel, subjects will be assigned randomly assigned Study ID numbers and then randomized using a computer-generated randomization algorithm. The research coordinator will record the randomization assignments on a

master list which will be maintained by the research coordinator on a password protected computer in a locked office. The research coordinator and research assistants will assemble the mailings based on this master list.

Subject Withdrawal
Subjects will be withdrawn from this study if they contact the study team and request to be withdrawn from this study. Withdrawal will not affect subject safety or the subject's care at Penn Family Care.

Subjects may withdraw from the study at any time without impact to their care. They may also be discontinued from the study at the discretion of the Investigator (e.g. subject self-reports previous CRC screening that meets the USPSTF CRC screening guidelines, subject contacts the study team and requests to be withdrawn from the study, etc.). The Investigator may also withdraw subjects to protect the subject for reasons of safety or for administrative reasons. It will be documented whether or not each subject completes the clinical study. No protected health information from PennChart will be maintained on behalf of this study. De-identified demographic information will be maintained for analysis purposes. The below table depicts the timeline of study enrollment described above.

	Pre-screening	Baseline<br>Day 0	FU to Mailing<br>Wk 3 (±3 days)	FU to Mailing<br>Wk 6 (±3 days)	FU to Mailing<br>Wk 9 (±3 days)
EMR review	X				
Randomization	X				
Mail FIT		X			
IVR			X		
Mailed reminder				X	
Questionnaire (subsample)					X

7. Analysis Plan

Power and Sample Size
Through the PFC quality improvement initiative, we have found the accuracy rate of the EMR algorithm to be approximately 80%. As such, we anticipate 1,080 of 1,350 subjects will be eligible **and randomized,** with 270 subjects enrolled on to each of the four arms in a 1:1:1:1 ratio. We estimate a base return rate for controls to be 15% ~~and a base return rate for the intervention arms to be 25%. We will compare the intervention arms against the control arms but we will not compare one intervention against another intervention.~~ **We will compare each intervention arm to the control and use Bonferroni correction for an alpha of .017 (.05/3) in order to account for three comparisons. Thus**, we have 80% power to detect an absolute ~~10%~~ **11 percentage point** increase in response rate using a two-sided Type 1 error rate of ~~0.05~~ **0.017**.

Data Analysis
We will conduct a chi-square analysis using statistical software Stata ~~12~~ to compare each intervention arm to the control arm~~. A second chi-square analysis will be performed by blinded members of the research team to compare response rates of the study participants and patients receiving FIT as standard clinical care.~~ Analysis will be conducted by the research team twelve weeks after the last subsample questionnaire is completed.

Subject Population(s) for Analysis
The population that will be included in the primary study analysis will include subjects who were randomized into the study and who completed FIT as an intention to treat analysis~~. Two populations will be included in the secondary analysis. The first will include any subjects randomly selected to receive a subsample questionnaire and who completed the questionnaire. The second will include patients who currently receive FIT as standard of care in the Penn Family Care Clinic. In order to provide a comparison group, retrospective chart review will be performed on this group in order to compare response rates and demographics between study participants and the eligible clinic population. The data collected on these patients will be identical to the data collected on study participants (i.e. demographics, inclusion criteria, exclusion criteria, response rates).~~

8. Data and Safety Monitoring

Protected Health Information/Data Protection
We will abstract the following variables from PennChart.

- Name
- Address
- Birth date
- Date of most recent CRC screening (e.g. colonoscopy, sigmoidoscopy, FOBT/FIT)

- Medical and family history
- Phone number
- Medical record number

Recording of Adverse Events
At each contact with the subject, the investigator will seek information on adverse events by specific questioning. Information on all adverse events will be recorded immediately in the source document, and also in the appropriate adverse event module of the case report form (CRF). All adverse events occurring during the study period will be recorded. The clinical course of each event will be followed until resolution, stabilization, or until it has been determined that the study intervention or participation is not the cause. Serious adverse events that are still ongoing at the end of the study period will be followed up to determine the final outcome. Any serious adverse event that occurs after the study period and is considered to be possibly related to the study intervention or study participation will be recorded and reported immediately. The relationship of each adverse event to the study procedures will be characterized by the PI and will be classified as either definitely related, probably related, unlikely to be related, or unrelated.

Reporting of Adverse Events, Adverse Device Effects and Unanticipated Problems
The PI and study team will conform to the adverse event reporting timelines, formats and requirements of the IRB and DSMC. If a narrative report is submitted, the following information will be provided to all reviewing entities:

Study identifier	Current status
Study Center	Whether study intervention was discontinued
Subject number	The reason why the event is classified as serious
A description of the event	Investigator assessment of the association between the
Date of onset	event and study intervention

Any other events will be recorded and reported in accordance with institutional and federal policies.

If an SAE has not resolved at the time of the initial report and new information arises that changes the investigator's assessment of the event, a follow-up report including all relevant new or reassessed information will be submitted to the IRB and DSMC. The PI will be responsible for ensuring that all SAE are followed until either resolved or stable.

Investigator Reporting: Notifying the Penn IRB
The investigator will submit reports to the IRB within ten working days (with one exception) of events that meet the definition of an unanticipated problem involving risks to subjects or others. Exception: If the adverse event involved a death and indicates that participants or others are at increased risk of harm, the investigator will submit a report to the IRB within three days.

The investigator will submit reports to the IRDSMC in accordance to their current reporting requirements:
All Grade 3 or higher events (AE or SAE) within five business days of knowledge.
All unexpected deaths within 24 hours of knowledge.
All others deaths within 30 days of knowledge. Deaths of subjects off-study for greater than 30 days from the last study treatment/intervention are not reportable.

Unblinding Procedures
Only the PI, co-investigator, and project manager will be blinded to what financial incentive subjects are randomized. If unblinding of members of the research team becomes necessary, we will document the following information in REDCap: why unblinding was necessary, date of approval to unblind from the IRB and DSMC, when unblinding occurred, and how unblinding occurred.

Confidentiality
Information about study subjects will be kept confidential and managed according to the requirements of the Health Insurance Portability and Accountability Act of 1996 (HIPAA). Please refer to our request to waive HIPAA authorization.

Data Collection and Management
Source documents are maintained in PennChart. No source documents will be printed or maintained in paper form at the study site. Data from PennChart will be recorded in Case Report Forms (CRFs) developed within Penn Medicine's REDCap system. The investigator and study team (which includes the project manager, research coordinator, and research assistants) will have access to PHI within PennChart and REDCap. We will label all PHI within REDCap as identifiable information so that de-identified exports are possible. All reports that include identifiable information will be stored on the Department of Family Medicine's secure drive, maintained behind the UPHS firewall. Once data analysis and manuscripts

have been published, the databases will be removed from REDCap and the data will be de-identified on the secure drive. This de-identified dataset will be stored for up to five years after analysis is complete and manuscripts have been published

Records Retention
Once analysis is completed and any manuscripts are published, we will retain personally identifiable information no longer than seven years in accordance with government regulations, applicable policies, and institutional requirements.

Study Monitoring Plan
The Principal Investigator will monitor this study with the research team on a bi-weekly basis. He will review the study progress through regular electronic and in person communication.

Auditing and Inspecting
At the discretion of the DSMC, this protocol will be audited approximately 12-18 months from the first subject enrolled and approximately every other year thereafter for the duration of the study. The Principal Investigator will be notified in advance of the selection of their protocol for review and the cases that have been randomly selected. Three randomly selected subjects or 10% of the total accrual, whichever is higher, will be audited. The committee may alter the frequency of re-monitoring based on the audit findings and degree of deficiencies.

The Principal Investigator will permit study-related monitoring, audits, and inspections by the DSMC and/or IRB, government regulatory bodies, and University compliance and quality assurance groups of all study related documents (e.g. source documents, regulatory documents, data collection instruments, study data etc.). The Principal Investigator will ensure the capability for inspections of applicable study-related facilities. As the Principal Investigator of this study, I accept the potential of inspection by government regulatory authorities and applicable University compliance and quality assurance offices.

9. Risk/Benefit Assessment

Risks
The risks associated with this study are no more than minimal. There is the potential risk of breach of confidentiality. We will minimize this risk by using de-identified information whenever possible and by maintaining all identifiable information on a secure drive and/or in a HIPAA-compliant system (e.g. REDCap). There is also the risk of psychological harm associated with being screened for cancer. We will minimize this risk by communicating the results of the screening test to the subject in a timely fashion and facilitating the scheduling of diagnostic testing if the screening test is positive.

Benefits
If a participant completes and returns the FIT, which is standard clinical care, the subjects will potentially benefit from participation by increasing the chances of identifying colorectal cancer at an early stage. Information learned from this study may benefit society through a better understanding of how to effectively increase the return rate of mailed CRC screening which could in turn increase the rate of CRC screening and reduce the rate of CRC mortality.

Risk Benefit Assessment
The risks associated with this study are no more than minimal. Better knowledge of how to increase mailed screening could potentially address one of the major barriers of accessing care, (i.e. having patients come in for clinical office visits). Additionally, FIT is less invasive than colonoscopy and, according to the USPSTF, considered equally effective if conducted once a year (as opposed to having a colonoscopy once every ten years). For these reasons and those outlined in the above benefits section, the Principal Investigator believes that the risks of participating in the study are outweighed by the potential benefits of participating in the study.

Summary of Protocol Changes Modifications

Protocol:
University of Pennsylvania Principal Investigator:

Date of Submission	Description of Modification	Rationale for Modification	Approval date
10/19/2015	Adding a study personnel: Jill Johnson	Change in study personnel	10/21/2015
11/5/2016	1- Created inserts to be mailed to subjects receiving financial incentive gift cards (arms 3 and 4),<br>2- Created negative results letter" to be mailed to subjects who have negative FIT and no active Penn Med Account,<br>3- Revised subsample questions to measure spending behavior of gift cards	Change in patient material	11/6/2015
12/10/2015	Insert letter for Arm 1 participants.<br>Adina moved to study contact (from key personnel)	Change in patient material<br>Change in study personnel	12/11/2015
1/19/2016	Revised initial invitation letter, removing: “You are receiving this test at no cost to you”<br>Created letter to mail to subjects who receive FIT in error	Change in patient material	1/28/2016
3/1/2016	Positive results letter created<br>Revised reminder letter (at 6 wks)	Change in patient material	3/7/2016
4/20/2016	Remove Adina and Kara, add Matt, change Jill's role and update email.<br>Upload C-2 form instruction document sent to lottery winner	Change in study personnel<br>Change in patient material	4/21/2016
8/1/2016	Add Rebecca as study contact	Change in study personnel	8/2/2016
8/19/2016	Add Cathy as key study personnel<br>Add one inclusion criteria "Does patient have a PCP within CPUP?"<br>Modified a second inclusion criteria requiring patients have at least two office visits in past two years<br>Included three exclusion criteria which exclude those with congestive heart failure, cirrhosis, and dementia.	Change in study personnel<br>Only include patients actively involved in Penn for care<br>Exclude patients with contraindications for diagnostic colonoscopy	8/23/2016
N/A	Add Mounika Kanneganti and Ece Unal as key study personnel	Change in study personnel	9/21/2016
10/21/2016	Add Aisha Salman as key study personnel<br>Updated incl/excl criteria, updated initial letter to indicate pts can ask their doctor about other screening options such as colonoscopy, updated all phone numbers in patient materials.	Change in study personnel<br>Administrative change	10/31/2016

11/1/2016	Update subsample questionnaire (content and phone numbers) and combined C-2 instructions with lottery gift card insert.	Administrative change	11/3/2016
11/8/2016	Change anemia criterion back to iron-deficient		11/14/2016
11/30/2016	Added comparison group of pts, updated estimated study duration, statistical software used and 6 mo. follow up. Also, fixed minor typos	Addition of study population	12/5/2016
1/27/2017	Add Aaron Ahn as key study personnel	Change in study personnel	1/30/2017
5/2/2017	Added the following to key study personnel: Nicole Gabler, Ndidi Enwereji, Tim McAuiffe, removed Ece Unal.<br>Added phone number to initial letter and updated subsample questionnaire	Change in study personnel<br>Administrative change	5/3/2017
5/22/2017	Remove Matthew Kearney from study contacts and remove Nicole Gabler from key study personnel to study contacts. Add Aisha Salman & Tory Harrington to key study personnel	Change in study personnel	5/23/2017
6/6/2017	Added Chandana Golla to key study personnel	Change in study personnel	6/7/2017
7/26/2017	Removed Chandana Golla from key study personnel, moved Nicole Gabler from study contact to key study personnel and add Ashley Woodards to study contact	Change in study personnel	7/30/2017
9/29/2017	Added Rachel Kasper as key study personnel	Change in study personnel	10/9/2017
10/11/2017	Remove Rachel Kasper from key study personnel and add Maximilian Herlim as key study personnel	Change in study personnel	10/16/2017
2/12/2018	Remove Mounika and Aaron as key study personnel. Ad Chelsea as key study personnel, add funding source.	Change in study personnel<br>Funding status change	2/19/2018
2/23/2018	Slim protocol	Administrative change	3/5/2018

Initial Statistical Analysis Plan

Power and Sample Size

Through the PFC quality improvement initiative, we have found the accuracy rate of the EMR algorithm to be approximately 80%. As such, we anticipate 1,080 of 1,350 subjects will be eligible with 270 subjects enrolled on to each of the four arms in a 1:1:1:1 ratio. We estimate a base return rate for controls to be 15% and a base return rate for the intervention arms to be 25%. We will compare the intervention arms against the control arms but we will not compare one intervention against another intervention. We have 80% power to detect an absolute 10% increase in response rate using a two-sided Type 1 error rate of 0.05.

Data Analysis

We will conduct a chi-square analysis using statistical software Stata 12 to compare each intervention arm to the control arm. A second chi-square analysis will be performed by blinded members of the research team to compare response rates of the study participants and patients receiving FIT as standard clinical care. Analysis will be conducted by the research team twelve weeks after the last subsample questionnaire is completed.

Subject Population(s) for Analysis

The population that will be included in the primary study analysis will include subjects who were randomized into the study and who completed FIT as an intention to treat analysis. Two populations will be included in the secondary analysis. The first will include any subjects randomly selected to receive a subsample questionnaire and who completed the questionnaire. The second will include patients who currently receive FIT as standard of care in the Penn Family Care Clinic. In order to provide a comparison group, retrospective chart review will be performed on this group in order to compare response rates and demographics between study participants and the eligible clinic population. The data collected on these patients will be identical to the data collected on study participants (i.e. demographics, inclusion criteria, exclusion criteria, response rates).

Final Statistical Analysis Plan

**New changes from initial protocol notated in bold, parts removed from initial protocol notated in strikethrough

Power and Sample Size

Through the PFC quality improvement initiative, we have found the accuracy rate of the EMR algorithm to be approximately 80%. As such, we anticipate 1,080 of 1,350 subjects will be eligible **and randomized,** with 270 subjects enrolled on to each of the four arms in a 1:1:1:1 ratio. We estimate a base return rate for controls to be 15% ~~and a base return rate for the intervention arms to be 25%. We will compare the intervention arms against the control arms but we will not compare one intervention against another intervention.~~ **We will compare each intervention arm to the control and use Bonferroni correction for an alpha of .017 (.05/3) in order to account for three comparisons. Thus**, we have 80% power to detect an absolute ~~10%~~ **11 percentage point** increase in response rate using a two-sided Type 1 error rate of ~~0.05~~ **0.017**.

Data Analysis

We will conduct a chi-square analysis using statistical software Stata ~~12~~ to compare each intervention arm to the control arm. ~~A second chi-square analysis will be performed by blinded members of the research team to compare response rates of the study participants and patients receiving FIT as standard clinical care.~~ Analysis will be conducted by the research team twelve weeks after the last subsample questionnaire is completed.

Subject Population(s) for Analysis

The population that will be included in the primary study analysis will include subjects who were randomized into the study and who completed FIT as an intention to treat analysis. ~~Two populations will be included in the secondary analysis. The first will include any subjects randomly selected to receive a subsample questionnaire and who completed the questionnaire. The second will include patients who currently receive FIT as standard of care in the Penn Family Care Clinic. In order to provide a comparison group, retrospective chart review will be performed on this group in order to compare response rates and demographics between study participants and the eligible clinic population. The data collected on these patients will be identical to the data collected on study participants (i.e. demographics, inclusion criteria, exclusion criteria, response rates).~~

Summary of Statistical Analysis Plan Modifications

The analysis plan was updated to include the following modification of the power calculation:

We will compare each intervention arm to the control and use Bonferroni correction for an alpha of .017 (.05/3) in order to account for three comparisons. Thus, we have 80% power to detect an absolute 10% 11 percentage point increase in response rate using a two-sided Type 1 error rate of 0.05 0.017.
