# Supplementary material for: Effect of Financial Incentives on Patient Use of Mailed Colorectal Cancer Screening Tests: A Randomized Clinical Trial
Source: JAMA Netw Open. 2019 Mar 22;2(3):e191156. doi: 10.1001/jamanetworkopen.2019.1156 (PMC6583304; doi:10.1001/jamanetworkopen.2019.1156)
Supplement: Supplement 2. — eTable. Inclusion and Exclusion Criteria for Data Extraction eFigure. Postintervention Interview Guide [file jamanetwopen-2-e191156-s002.pdf]

Supplementary Online Content

Mehta SJ, Pepe RS, Gabler NB, et al. Effect of financial incentives on patient use of mailed colorectal cancer screening tests: a randomized clinical trial. *JAMA Netw Open*. 2019;2(3):e191156.
doi:10.1001jamanetworkopen.2019.1156

eTable. Inclusion and Exclusion Criteria for Data Extraction
eFigure. Postintervention Interview Guide

This supplementary material has been provided by the authors to give readers additional information about their work.

© 2019 Mehta SJ et al. *JAMA Network Open*.

eTable. Inclusion and Exclusion Criteria for Data Extraction

**Variable**	**Data Field**	**Criteria**
**Inclusion**		
Age	Age, DOB	50 through 74 years
Has received care at Penn Family Care	Encounters<br>Office Visit to Penn Family Care (PPMC, FMCH)	At least two office visit to PFC within the past 2 years
Due for Colorectal cancer screening	Health maintenance tab (colorectal cancer)	Overdue status of CRC screening tab
PCP is a CPUP doctor	PCP	PCP must be one of the following providers:
**Exclusion**<br>***Patients meeting one or more of these criteria NOT eligible to be pulled*		
Colonoscopy completed within 10 years	Procedures, diagnostic/procedure codes, billing codes	**CPT**: 44388-44397, 45355, 45378-45387, 45391, 45392<br>**HCPCS:** G0105, G0121.<br>**ICD-9 proc**: 45.21-5, 45.42-3
Sigmoidoscopy within the past 5 years	Procedures, diagnostic/procedure codes, billing codes	**ICD-9 proc:** 45.24, 48.21-4, 48.36<br>**CPT**: 45300, 45303, 45305, 45307-45309, 45315, 45317, 45320-1, 45327, 45355, 45387, 45388, 45391-45392, 45330-45335, 45337-45342, 45345
FOBT/FIT within the past year	Labs, diagnostic/procedure codes, billing codes	**CPT**: 82270-82274<br>**HCPCS:** G0328<br>**LOINC:** 2335-8, 12503-9, 12504-7, 14563-1, 14564-9, 14565-6, 27396-1, 27401-9, 27925-7, 27926-5, 29771-3, 56490-6, 56491-4, 57905-2, 58453-2
Has a history of colorectal cancer or polyps	Problem list, diagnostic/procedure codes, billing codes	**ICD-9**: 153.1, 153.2, 153.3, 153.6, 153.9, 209.13, 209.14, 209.15, 209.16, 209.53, 209.54, 209.55, 209.56, 211.3, 230.3, 556.4, V10.05-V10.06, V12.72<br>**ICD-10:** C18-C20, C21.2, C21.8, Z85.038, Z85.048<br>**HCPCS:** G0213-G0215, G0231

© 2019 Mehta SJ et al. *JAMA Network Open*.

Has history of other Gastrointestinal cancer	Problem list, diagnostic/procedure codes, billing codes	**ICD-9:** V10.00-V10.04, V10.07-V10.09<br>**ICD-10**: Z85 (Z85.00-Z85.09)
Has history of confirmed IBD (e.g. Crohn's Disease, ulcerative colitis)	Problem list, diagnostic/procedure codes, billing codes	**ICD-9:** 556.5, 556.6, 556.8, 556.9, 555.0, 555.1, 555.2, 555.9<br>**ICD-10:** K51.80, K51.811, K51.812, K51.813, K51.814, K51.818, K51.819, K51.90, K51.911, K51.912, K51.913, K51.914, K51.918, K51.919, K50.00, K50.011, K50.012, K50.013, K50.014, K50.018, K50.019, K50.10, K50.111, K50.112, K50.113, K50.114, K50.118, K50.119, K50.80, K50.811, K50.812, K50.813, K50.814, K50.818, K50.819, K50.90, K50.911, K50.912, K50.913, K50.914, K50.918, K50.919
Has a history of colitis other than Crohn's disease or ulcerative colitis	Problem list, diagnostic/procedure codes, billing codes	**ICD-9:** 006.2, 009.0, 009.1, 558.1, 558.2, 558.3, 558.42, 558.9<br>**ICD-10**: A06.2, A09, K51.50, K51.511, K51.512, K51.513, K51.514, K51.518, K51.519, K52.0, K52.1, K52.2, K52.82, K52.89, K52.9
Family History of CRC or polyps	Family History, diagnostic/procedure codes, billing codes	**ICD-9**: V16.0, V18.51<br>**ICD-10**: Z80.0, Z83.71
Has had colectomy	Problem list, procedures, diagnostic/procedure codes, billing codes	**CPT**: 44140, 44141, 44143, 44144, 44145, 44146, 44147, 44150-44153, 44155, 44156,44157,44158, 44160, 44210-44212<br>**ICD-10**: ODTE0ZZ, ODTE4ZZ, ODTE7ZZ, ODTE8ZZ
Has been diagnosed with Lynch Syndrome (HNPCC)	Problem list, diagnostic/procedure codes, billing codes	**ICD-9:** V18.9, V84.09<br>**ICD-10**: Z84.81, Z15.09
Has been diagnosed with Familial Adenomatous Polyposis (FAP)	Problem list, diagnostic/procedure codes, billing codes	**ICD-9:** 211.3<br>**ICD-10**: D12.0, D12.1, D12.6, D63.5
Has anemia, iron deficient	Problem list, diagnostic/procedure codes, billing codes	**ICD-10:** D50, D50.0, D50.1, D50.8, D50.9, Z86.2

Has a history of lower GI bleeding	Problem list, diagnostic/procedure codes, billing codes	**ICD-9**: 578, 578.0, 578.1, 578.9<br>**ICD-10**: K92.0
Has metastatic (stage IV) blood or solid tumor cancer	Problem list, diagnostic/procedure codes, billing codes	**ICD-10**: C79.89, C80.1<br>**ICD-9**: 198.89, 199.1
Has end stage renal disease	Problem list, diagnostic/procedure codes, billing codes	**ICD-10**: E11.22, I12.0, I13.0, I13.11, I13.2, I50.9, N18.1, N18.2, N18.3, N18.4, N18.5, N18.6, N18.9, Z99.2<br>**ICD-9**: 403.01, 403.11, 403.91, 404.02, 404.03, 404.12, 404.13, 404.92, 404.93, 585.6
Has congestive heart failure (CHF)	Problem list, diagnostic/procedure codes, billing codes	**ICD-10:** I11.0, I12.0, I13.0, I13.2, I38, I50.1, I50.20, I50.21, I50.22, I50.23, I50.30,<br>I50.31, I50.32, I50.33, I50.40, I50.41, I50.42, I50.43, I50.9, N18.1, N18.3, N18.4, N18.5, N18.6, N19, Z86.79<br>**ICD-9:** 428.1, 428.2, 428.3, 428.4, 428.9
Has end-stage liver disease/Cirrhosis	Problem list, diagnostic/procedure codes, billing codes	**ICD-10:** E83.110, K70.30, K71.7, K74.3, K74.4, K74.5, K74.60, K74.69, P78.81, Z83.79, Z87.19<br>**ICD-9:** 571.5, 571.9
Has dementia	Problem list, diagnostic/procedure codes, billing codes	**ICD-10:** A81.0, A81.01, B20, F01.50, F01.51, F02.80, F02.81, F03.90, F03.91, F05, F06.8, F10.27, F10.97, F19.27, F19.97, F22, F32.9, G10, G12.21, G30.0, G30.1, G30.9, G31.09, G31.83, R41.0, S09.90XA<br>**ICD-9:** 290, 291.10, 290.11, 290.12, 290.13, 290.2, 290.21, 290.3, 290.4, 290.41<br>290.42, 290.43, 291, 291.9<br>292.82, 294.1, 294.2, 294.21

© 2019 Mehta SJ et al. *JAMA Network Open*.

eFigure. Postintervention Interview Guide

This survey is a part of a research study to learn about the best ways to get people screened for colon cancer. I will ask you a few questions about you and your experience with colon cancer screening; it will take 5 to 10 minutes. Your participation is voluntary and your answers will remain confidential. If at any time you feel uncomfortable, you can skip questions or tell me that you no longer want to participate, with no penalty. Your answers will teach us about how we can get more patients screened for colon cancer. If you have any questions, feel free to ask me now or call us at xxx-xxx-xxxx. Do you agree to participate?

1.) As a part of a new program, some patients at Penn Family Care are sent a "FIT" kit in the mail. It is an at-home screening test for colon cancer where you send a swab of stool in to the lab to test for blood. Most doctors want their patients who are 50-75 to either do this test every year or get a colonoscopy once every ten years. Do you remember being sent a test like this?

2.) Before being invited to participate in this program, had you ever been screened for colon cancer with any type of test? Tests you may have had include: Colonoscopy, Flexible Sigmoidoscopy, or a Stool test (guaiac-based FOBT or FIT).

a. [If yes] Which test? Do you remember when? Where did you get it done?

3.) In the future, would you prefer to have a colonoscopy every 10 years or complete a FIT every year?

a. FIT
b. Colonoscopy
c. No preference
d. Prefer not to be screened

4.) Can you tell me more about why this is your preference?

5.) If you could choose, how would you want to be reminded to get screened for colon cancer?

6.) Generally speaking, what do you think about receiving an at-home colon cancer screening test in the mail?

7.) What might motivate you to get screened for colon cancer in the future?

The last few questions are a bit personal, so let me know if you'd like to skip any of them.

8.) What is your marital status?

A. Single (Never married)
B. Married/ Domestic Partnership
C. Separated / Divorced
D. Widowed

© 2019 Mehta SJ et al. *JAMA Network Open*.

E. Separated
F. Prefer not to answer

9.) What is the highest grade or year of school you completed?
A. Some high school
B. High school graduate/GED
C. Some college / vocational / trade school
D. Graduate of college / vocational/ trade school
E. College grad plus
F. Prefer not to answer

10.) What is your current employment status?
A. Employed
B. Unemployed
C. Homemaker
D. Student
E. Retired
F. Unable to work
G. Don’t know / unsure
H. Prefer not to answer

11.) What is your annual household income from all sources?
A. Less than $25,000
B. $25,000 to less $50,000
C. $50,000 to less than $70,000
D. $70,000 or more
E. Don’t know / not sure
F. Prefer not to answer

Thank you for taking the time to complete this survey.

We appreciate your feedback.

© 2019 Mehta SJ et al. *JAMA Network Open*.
