# Supplementary material for: Effect of Financial Incentives on Patient Use of Mailed Colorectal Cancer Screening Tests: A Randomized Clinical Trial
Source: JAMA Netw Open. 2019 Mar 22;2(3):e191156. doi: 10.1001/jamanetworkopen.2019.1156 (PMC6583304; doi:10.1001/jamanetworkopen.2019.1156)
Supplement: Supplement 3. — Data Sharing Statement [file jamanetwopen-2-e191156-s003.pdf]

Data Sharing Statement

Mehta. Effect of Financial Incentives on Patient Use of Mailed Colorectal Cancer Screening Tests. *JAMA Netw Open*. Published March 22, 2019. 10.1001/jamanetworkopen.2019.1156

Data
Data available: No

Additional Information
Explanation for why data not available: Sharing of data was not part of the protocol approved by the Institutional Review Board
